# Supplementary material for: Neurological Manifestations in Pediatric COVID-19 Patients Hospitalized at King Abdulaziz University Hospital, Jeddah, Saudi Arabia: A Retrospective Study
Source: Children (Basel). 2022 Nov 30;9(12):1870. doi: 10.3390/children9121870 (PMC9776438; doi:10.3390/children9121870)
Supplement: Supplementary file 1 [file children-09-01870-s001.zip › children-2064510-supplementary.pdf]

Supplementary Materials

Table S1. Additional demographic and clinical data of the whole studied group (N=94)

Variables	Values
**Age** Median, IQR, Range (mo)	78 (11.25-192), (0.16-228)
**Age category**<br>*Neonate*<br>*Infant*<br>*Child*<br>*Adolescent*	**N** **%**<br>10 10.6<br>17 18.1<br>33 35.1<br>34 36.2
**Duration from appearance of symptoms till diagnosis and admission** Median, IQR, Range (Days)	2 (2-4), (1-15)
**Duration form appearance of symptoms till development of neurologic manifestations** Median, IQR, Range (Days)	1 (1-3), (1-6)
**Length of hospital stay:** Median, IQR, Range (days)	4 (3-6), (1-54)

Table S2. Vital signs and weight for age and sex of the whole studied group (N=94)

Variable	(Mean ± SD, Range)	
**Temperature (^{0} C)**	37.35 ± 0.85 (36.2-40)	
**Heart rate (beats/min)**	113.69 ± 30.88 (66-203)	
**Respiratory rate (breaths/min)**	26.84 ± 9.15 (17-68)	
**O2 saturation (Pulse oximetry)**	97.04 ± 3.27 (83-100)	
**Blood pressure for age and sex**	**N**	**%**
Normal	64	68.1
Low	29	30.9
High	1	1.1
**Weight for age and sex**	**N**	**%**
Normal	86	91.5
Low	8	8.5

Table S3. Comparison of additional clinical data between pediatric COVID-16 cases with and without neurologic manifestations

Variables	COVID-19 cases without neurologic manifestations N=65		COVID-19 cases with neurologic manifestations N=29		Significance p value
**Age (Median, IQR, Range) (mo)**	60 (12-186) (0.16-228)		132 (7-204) (0.4-228)		0.35 [a]
	N	%	N	%	
Neonate	6	9.2	4	13.8	
Infant	12	18.5	5	17.2	0.44 [b]
Child	26	40.0	7	24.1	
Adolescent	21	32.3	13	44.8	
**Duration from appearance of symptoms till diagnosis and admission** Median, IQR, Range (Days)	2 (2-3), (1-15)		2 (2-6), (1-15)		0.22 [a]
**Duration form appearance of symptoms till COVID-19 confirmed diagnosis** Median, IQR, Range (Days)	2 (1-3), (1-15)		3 (1-5), (1-15)		0.08 [a]
**Length of stay: Median, IQR, Range (days)**	4 (3-5.5) (1-54)		5 (2.5-9) (1-40)		0.25 [a]

[a] Mann Whitney U test, [b] Chi square test

Table S4. Comparison of vital signs and weight for age and sex between pediatric COVID-16 cases with and without neurologic manifestations

Variables	COVID-19 cases without neurologic manifestations N=65		COVID-19 cases with neurologic manifestations N=29		Test value	Significance p value
**Temperature (^{0} C)**	37.35 ± 0.89 (36.2-40.0)		37.36 ± 0.79 (36.2-39.3)		t=0.33	0.97 [a]
**Heart rate (beats/min)**	114.17 ± 27.78 (72-180)		112.62 ± 37.44 (66-203)		t=0.22	0.82 [a]
**Respiratory rate (breaths/min)**	27.14 ± 9.66 (17-68)		26.17 ± 8.02 (17-42)		t= 0.47	0.64 [a]
**O2 saturation (Pulse oximetry)**	97.18 ± 2.97 (83-100)		96.72 ± 3.88 (84-100)		t=0.63	0.53 [a]
**Blood pressure for age and sex**						
Normal	46	70.8	18	62.1	X^2=2.64	0.27 [b]
Low	19	29.2	10	34.5		
High	0	0	1	3.4		
**Weight for age and sex**						
Normal	58	89.2	28	96.6	X^2=1.38	0.24 [b]
Low	7	10.8	1	3.4		

[a] Independent t-test, [b] Chi square test

Table S5. Comparison of categorial variables of laboratory and radiological findings between pediatric COVID-19 cases with and without neurological manifestations

Variables (measured value)	COVID-19 cases without neurologic manifestations N=65			COVID-19 cases with neurologic manifestations N=29			Significance p-value
	**Category**	**N**	**%**	**Category**	**N**	**%**	
Red blood cells (Mean ±SD), range ($x10^6/mm^3$)	Normal RBC count	35	53.8	Normal RBC count	21	72.4	0.09 [c]
	Low (anemia)	28	43.1	Low (anemia)	6	20.7	
	High (polycythemia)	2	3.1	High (polycythemia)	2	6.9	
Hemoglobin (HB) (Mean ± SD), (gm/dl)	Normal HB	34	52.3	Normal HB	17	58.6	0.70 [c]
	Low HB	28	43.1	Low HB	10	34.5	
	High HB	3	4.6	High HB	2	6.9	
White blood cells (Median, IQR) ($x10^3/mm^3$)	Normal WBC count	50	76.9	Normal WBC count	20	69	0.46 [c]
	Leucopenia	4	6.2	Leucopenia	4	13.8	
	Leukocytosis	11	16.9	Leukocytosis	5	17.2	
Neutrophils (Median, IQR) ($x10^3/mm^3$)	Normal count	54	83.1	Normal count	23	79.3	0.62 [c]
	Neutropenia	7	10.7	Neutropenia	5	17.2	
	Neutrophilia	4	6.2	Neutrophilia	1	3.4	
Lymphocytes (Median, IQR) ($x10^3/mm^3$)	Normal count	44	67.7	Normal count	18	62.1	0.60 [c]
	Lymphopenia	21	32.3	Lymphopenia	11	37.9	
Platelet count (Median, IQR) ($x10^3/mm^3$)	Normal count	55	84.6	Normal count	26	89.7	0.73 [c]
	Thrombocytopenia	5	7.7	Thrombocytopenia	2	6.9	
	Thrombocytosis	5	7.7	Thrombocytosis	1	3.4	
CRP (Median, IQR) (mg/dl)	Normal	47	72.3	Normal	20	69	0.74 [c]
	High	18	27.7	High	9	31	
Ferritin (Median, IQR) (ng/ml)	Normal	53	81.5	Normal	22	75.9	0.53 [c]
	High	12	18.5	High	7	24.1	
D-dimer (Median, IQR) ug/ml	Normal	44	67.7	Normal	17	58.6	0.39 [c]
	High	21	32.3	High	12	41.4	
INR (Mean ± SD)	Normal	58	89.2	Normal	25	86.2	0.67 [c]
	High	7	10.8	High	4	13.8	
APTT (Mean ± SD) Seconds	Normal	63	96.9	Normal	26	89.7	0.15 [c]
	High	2	3.1	High	3	10.3	
Alanine aminotransferase (Median, IQR) (U/L)	Normal	57	87.7	Normal	21	72.4	0.07 [c]
	High	8	12.3	High	8	27.6	
Aspartate aminotransferase (Median, IQR) (U/L)	Normal	45	69.2	Normal	20	69	0.98 [c]
	High	20	30.8	High	9	31	
Total serum bilirubin (Median, IQR) (mg/dl)	Normal	55	84.6	Normal	27	93.1	0.25 [c]
	High	10	15.4	High	2	6.9	
Serum albumin (Median, IQR) (gm/L)	Normal	58	89.2	Normal	24	82.8	0.39 [c]
	Low	7	10.8	High	5	17.2	
BUN (Median, IQR) (mmol/L)	Normal	59	90.8	Normal	25	86.2	0.51 [c]
	High	6	9.2	High	4	13.8	
Serum creatinine (Median, IQR) (umol/L)	Normal	57	87.7	Normal	23	79.3	0.29 [c]
	High	8	12.3	High	6	20.7	
**Radiological findings (Chest X-ray)**	Normal	55	84.6	Normal	23	79.3	0.53 [c]
	Abnormal infiltrations	10	5.4	Abnormal infiltrations	6	20.7	

[c] Chi square test
